# Supplementary material for: Cellulose Modification for Improved Compatibility with the Polymer Matrix: Mechanical Characterization of the Composite Material
Source: Materials (Basel). 2020 Dec 3;13(23):5519. doi: 10.3390/ma13235519 (PMC7729504; doi:10.3390/ma13235519)
Supplement: Supplementary file 1 [file materials-13-05519-s001.pdf]

materials

Supplementary Materials

Cellulose Modification for Improved Compatibility with the Polymer Matrix: Mechanical Characterization of the Composite Material

Stefan Cichosz, Anna Masek and Adam Rylski

1. Examplary Stress-strain Curves

Below, exemplary stress-strain curves for the composite sample filled with ND/MA/1/E at the loading level of 35 wt% are presented:

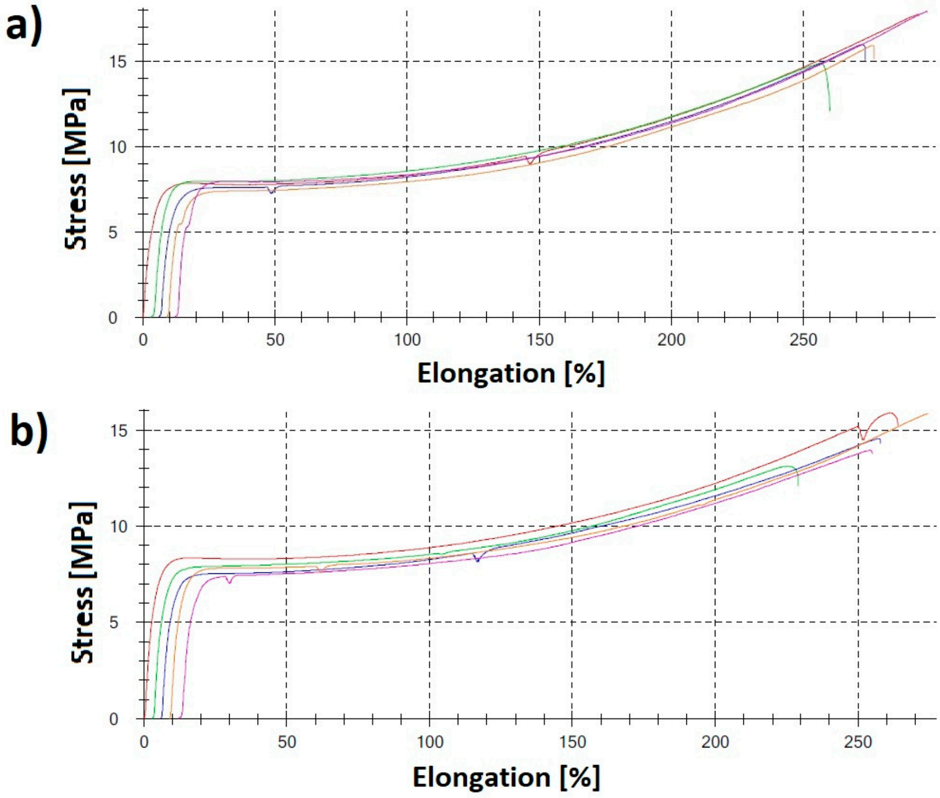

Figure S1. Stress-strain curves of sample filled with ND/MA/1/E at loading of 35 wt%: (**a**) in vertical, (**b**) in horizontal direction.

© 2020 by the authors. Licensee MDPI, Basel, Switzerland. This article is an open access article distributed under the terms and conditions of the Creative Commons Attribution (CC BY) license (http://creativecommons.org/licenses/by/4.0/).
